# Identifying MicroRNAs Involved in Aging of the Lateral Wall of the Cochlear Duct

**DOI:** 10.1371/journal.pone.0112857

**Published:** 2014-11-18

**Authors:** Qian Zhang, Huizhan Liu, Garrett A. Soukup, David Z. Z. He

**Affiliations:** Department of Biomedical Sciences, Creighton University School of Medicine, Omaha, Nebraska, United States of America; University of Salamanca- Institute for Neuroscience of Castille and Leon and Medical School, Spain

## Abstract

Age-related hearing loss is a progressive sensorineural hearing loss that occurs during aging. Degeneration of the organ of Corti and atrophy of the lateral wall of the cochlear duct (or scala media) in the inner ear are the two primary causes. MicroRNAs (miRNAs), a class of short non-coding RNAs that regulate the expression of mRNA/protein targets, are important regulators of cellular senescence and aging. We examined miRNA gene expression profiles in the lateral wall of two mouse strains, along with exploration of the potential targets of those miRNAs that showed dynamic expression during aging. We show that 95 and 60 miRNAs exhibited differential expression in C57 and CBA mice during aging, respectively. A majority of downregulated miRNAs are known to regulate pathways of cell proliferation and differentiation, while all upregulated miRNAs are known regulators in the pro-apoptotic pathways. By using apoptosis-related gene array and bioinformatic approaches to predict miRNA targets, we identify candidate miRNA-regulated genes that regulate apoptosis pathways in the lateral wall of C57 and CBA mice during aging.

## Introduction

Age-related hearing loss (ARHL), also known as presbycusis, is a progressive sensorineural hearing loss. It has been reported that as many as 30 to 35% of the population aged between 65 and 75 have ARHL [1], [2]. The prevalence and severity of hearing loss are much higher in populations older than 80 [3]. A recent study shows that more than 95% of centenarians suffer from severe to profound hearing loss [4]. Schuknecht classified the etiology of presbycusis into six distinct causes [5]. The two major causes are due to degeneration of the organ of Corti (OC) and the lateral wall (LW) of the scala media, which includes the stria vascularis (SV) and the spiral ligament [6], [7]. The OC contains mechanosensitive hair cells that transduce mechanical vibration into electrical signal, while SV pumps potassium ions to the scala media and generates endocochlear potential (EP), which is essential for hair cell mechanotransduction. Stria-originated ARHL is usually characterized by reduction of EP and atrophy/degeneration of the LW.

MicroRNAs (miRNAs) are a class of post-transcriptional regulators. They are short ∼22 nucleotide RNA sequences that bind to complementary sequences in the 3′ UTR of multiple target mRNAs, usually resulting in their silencing. miRNAs, collectively targeting ∼60% of all genes, are abundantly present in all human cells and able to repress hundreds of target genes each [8], [9], [10], [11]. miRNAs are required for the fine-tuning and tight regulation of a wide range of cellular processes and biological functions, including cell differentiation, proliferation, apoptosis, mobility, migration, metabolism, and self-renewal [12]. Recent studies have established a direct correlation between miRNA regulation and aging in worms (*Caenorhabditis elegans*), mice, and humans [13], [14]. Several miRNAs have been implicated in the occurrence and progression of age-related diseases, such as Alzheimer's disease, type II diabetes, pulmonary heart disease, and cardiovascular diseases [15], [16].

Since miRNA expression is cell/tissue-specific and time-dependent, which is consistent with the specific functions of different signaling pathways in different cells/tissues at different stages during development and aging [17], the analysis of miRNAs that are differentially expressed with aging has led to the discovery of numerous miRNAs that are important for controlling aging processes [13], [18], [19]. By comparing the expression profiles of miRNAs in the OC from younger and old mice, we identified approximately 100 miRNAs that exhibited differential expression, with downregulated miRNAs significantly outnumbering upregulated miRNAs [20]. The goal of the current study was to identify miRNAs that are differentially expressed in the LW during aging and to explore their potential apoptosis-related target genes. We first examined strial morphology and measured EP at different stages during aging from two mouse strains, C57BL/6J and CBA/J. We then used GeneChip microarray technology to identify differentially expressed miRNAs in the LW. Quantitative real-time PCR (q-PCR) and *in situ* hybridization techniques were used to determine the temporal and spatial expression of several subsets of miRNAs identified by the microarray analysis. We subsequently used a quantitative PCR array to examine apoptosis-related gene expression in the LW. Finally, we used target prediction algorithms and bioinformatics tools to explore potential regulatory networks of apoptosis signaling pathways composed of miRNAs and mRNAs in the LW.

## Materials and Methods

### SV tissue collection and RNA extraction

C57BL/6J and CBA/J mice were bred in-house, with breeding pairs purchased from the Jackson Laboratory (Bar Harbor, ME, USA). Care and use of the animals in this study were approved by the Creighton University Institutional Animal Care and Use Committee.

Cochleae were rapidly dissected in cold phosphate-buffered saline (PBS) with 10 mM Na_2_HPO_4_, 1.7 mM KH_2_PO_4_, 137 mM NaCl, 2.7 mM KCl, and pH 7.4. The LW of scala media from all cochlear turns was isolated. Ten cochleae from five mice were pooled to generate each sample, and three independent samples were prepared for triplicate GeneChip miRNA array analyses. The animal ages for microarray analysis were postnatal 21 days (P21), 9 months (9 m) and 16 months (16 m) for both strains of mice. The isolated LW tissue was stored at −20°C in RNAlater stabilization reagent (Ambion, Austin, TX, USA). Total RNA including miRNAs was isolated using mirVana miRNA Isolation Kit (Ambion) and dissolved in 20–30 µl of RNase free water. RNA concentration was determined by UV spectrophotometry (Nanodrop ND-1000), and the quality of each RNA sample was verified by measuring the ratio of 28*S* to 18*S* rRNA using an Agilent 2100 BioAnalyzer. Additional samples of total RNA from the LW were prepared for quantitative PCR assays using the same procedure.

### Microarray analysis of miRNAs

The miRNA expression profile for each total RNA sample of the LW was determined using GeneChip miRNA 1.0 arrays (Affymetrix, Santa Clara, CA, USA). Synthesis of cDNA, hybridization to chips, and washes were performed according to the manufacturer's protocol. GeneChip arrays were scanned at 3-µm density using a GeneArray Scanner (Affymetrix). Images were inspected to ensure that all 18 chips had low background and bright hybridization signals. Mean signal fluorescence signal intensity for each probe was quantile normalized. Each miRNA probe was assessed for detection based on a Wilcoxon Rank-Sum test of the miRNA probe set signals compared to the distribution of signals from the background. One-way ANOVA test was used to determine significant differences in miRNA expression between P21 and older age groups, where p<0.05 was interpreted as significant. The raw data of the miRNA array has been submitted to the National Center for Biotechnology Information-Gene Expression Omnibus (GEO) (GEO submission number: GSE57782).

### Quantitative real-time PCR

For q-PCR analysis of miRNAs, the LW from five additional animals was obtained. Q-PCR detection of miRNAs was performed using mirVana q-PCR miRNA Primer sets (Ambion). 100 ng total RNA from each group was reverse transcribed using SuperScript Reverse Transcriptase (Invitrogen) in a 20 µl reaction to produce cDNA. q-PCR was performed using a 7500 Fast Real-Time PCR System (Applied Biosystems) to analyze triplicate reactions (20 µl) containing 2× SYBR Green PCR Master Mix (Applied Biosystems), a 10-fold dilution of mirVana q-PCR Primer Set, and cDNA. After incubation at 95°C for 20 s, PCR products were analyzed throughout 40 cycles consisting of incubation at 95°C for 3 s and 60°C for 30 s. U6 RNA was detected as an internal relative control for each group. The threshold cycle (C_T_) was defined as the PCR cycle number at which the fluorescence intensity was appreciably above the background level but was still in the early exponential phase of amplification. The change in C_T_ between a given miRNA and U6 RNA for each reaction was defined as ΔC_T_ and averaged for each group. The change in ΔC_T_ between two groups was defined as ΔΔC_T_, which represents the relative difference in expression of miRNA. Fold difference in miRNA expression between groups was calculated as 2^-ΔΔCT^. We compared the ΔC_T_ values for each miRNA from older groups with those for the P21 group using the one-way ANOVA test, where p<0.05 was interpreted as significant.

### In Situ Hybridization

Control digoxigenis (DIG)-labeled beta-actin mRNA riboprobe (633 nt) was prepared by *in vitro* transcription from DNA templates using T7 RNA polymerase and DIG-UTP (Roche) and purified using RNAeasy spin columns (Qiagen). DNA templates for *in vitro* transcription of the beta-actin riboprobe were generated by PCR using primers 5′-TAATACGACTCACTATAGGG ACCGCTCGTTGCCAATAGTG and 5′-TGGTGGGAATGGGTCAGAAG and random-hexamer-primed mouse cDNAs.

Cross-section preparations were used for *in situ* hybridization as previously described [21], [22], [23]. Anesthetized mice were transcardially perfused with PBS followed by PBS containing 4% PFA. Inner ears were dissected, fixed in PBS containing freshly dissolved 4% paraformaldehyde (PFA), and decalcified with 0.5M EDTA for 24–48 h. The cochleae were cryoprotected and embedded in cutting temperature compound (O.C.T. Tissue-Tek, Sakura Finetek, Torrance, CA) for frozen sectioning. After incubated in PBS with diluted Proteinase K (Sigma catalog # P4850) and defatted through a graded ethanol series and rehydrated in PBS containing 0.1% TWEEN 20, the serial cross-sections (6 µm) were pre-hybridized in 2 mL hybridization solution (50% formamide, 2× SSC, 0.1% Tween 20, and 150 µg/ml denatured herring sperm DNA) for 1–3 h at 53°C. Hybridizations were incubated overnight at 53°C following the addition of 12 pmol DIG-labeled LNA probe or 1 pmol DIG-labeled beta-actin mRNA riboprobe denatured for 10 min at 68°C in hybridization buffer. Tissues were washed at 50°C in 0.5× SSC, and RNA was digested by incubation for 1 h at 37°C with RNase A (100 mM Tris–HCl pH 7.5, 500 mM NaCl, 0.1% TWEEN 20, and 10 µg/ml RNase A). After the digestions were terminated, tissues were incubated in wash and block for 1 h at 4°C and incubated overnight in a 1000-fold dilution of sheep anti-DIG-AP Fab fragment (Roche Diagnostics Corporation, Indianapolis, IN) in block buffer. Samples were washed at 4°C with wash buffer, and alkaline phosphatase activity was detected using BM Purple AP Substrate (Roche) by incubation for 2–16 h at 25°C. Reactions were terminated for at least 1 h in PBS (pH 5.5) containing 10 mM EDTA. Tissues were fixed by incubation overnight at 4°C in PBS containing 4% PFA. Fixed tissues were mounted in glycerol under glass cover slips for image acquisition as described previously [24]. Localization of BM purple was visualized by differential interference contrast optics.

### q-PCR Array of Apoptosis-Related Genes

Total RNA isolated from LW of each type of mouse at two ages was used to examine apoptosis-related gene expression by reverse transcription and RT-PCR using an RT2 Profiler q-RT-PCR Array (SABiosciences, Frederick, MD, USA). The 84 apoptosis-related genes contained in the array are listed in Table S1. Details of the procedures were provided in a previous study [25]. Upon completion of total RNA extraction from the LW and quality assessment, first strand cDNA was synthesized using oligodT-primed reverse transcription supplied with the RT2 first strand kit (SABiosciences). This kit contains genomic DNA elimination buffer and a built-in external RNA control. Total RNA used for cDNA synthesis was 500 ng for each sample. Expression of 84 apoptosis-related genes was evaluated in the LW tissue at the ages of P21 and 16 month in a 96-well plate (including five housekeeping and seven control genes; Mouse Apoptosis RT2 Profiler PCR Array, SABiosciences, PAMM-012C). RT^2^Real-TimeTM SYBR Green/fluorescein PCR Master Mix was used to monitor the fluorescence signal during each cycle of PCR using a 7500 Fast Real-Time PCR Detection System (Applied Biosystems). Each reaction started with an initial denaturation cycle at 95°C for 10 min, followed by 40 cycles consisting of 15 s at 95°C, and 1 min at 60°C for annealing. Each sample was repeated three times. Upon completion of the PCR, threshold cycle (C_T_) values were calculated automatically. The C_T_ value of each apoptosis-related gene was normalized by the average C_T_ value of five housekeeping genes to determine ΔC_T_. The change in ΔC_T_ for each gene between groups (ΔΔC_T_) represents the relative difference in expression, and fold difference was calculated as 2^-ΔΔCt^. One-way ANOVA test was used to compare ΔC_T_ values for each gene from older groups with those for the P21 group, where p<0.05 was interpreted as significant.

### miRNA Target Prediction of Apoptosis Related Genes

The potential mRNA targets of differentially expressed miRNAs were predicted by the TargetScan mouse version 6.2 (http://www.targetscan.org/mmu_61/). Analyses were based on expression profiles from GeneChip microarray and apoptosis q-PCR array. Both conserved and non-conserved targets for conserved miRNA families were considered. TargetScan predictions were used to construct a bipartite network graph in which miRNAs were connected to their predicted targets.

### Recording of EP

Animal were anesthetized with a combined regimen of ketamine (16.6 mg/ml) and xylazine (2.3 mg/ml) and supplemented as needed to maintain a surgical level of anesthesia. An incision was then made in the inferior portion of the right postauricular sulcus. The bulla was perforated allowing for exposure of the stapedial artery, basal turn, and upper turn of the cochlea. A basal turn location was chosen to record EP. This location was approximately 2 mm from the basal end, corresponding to the region with best frequency of 20–30 kHz, based on the cochlear frequency map for the mouse [26]. A hole was made using a fine drill. A glass capillary pipette electrode (10–20 MΩ) filled with 150 mM KCl was mounted on a hydraulic micromanipulator and advanced until a stable positive potential was observed. The ground electrode was implanted in the dorsal neck muscles. The biological signals (filtered at 1 kHz) were amplified under current-clamp mode using an Axopatch 200B amplifier (Molecular Devices, Sunnyvale, CA, USA) and acquired by software pClamp 9.2 (Molecular Devices) running on an IBM-compatible computer with a 16-bit A/D converter (Digidata 1322B, Molecular Devices). The voltage changes during a penetration were continuously recorded under the Gap-free model using the Clampex in the pClamp software package. The sampling frequency was 10 kHz. Data were analyzed using Clampfit and Igor Pro (WaveMetrics, Portland, OR, USA).

### Morphology of LW

Cochleae were isolated at the age of P21, 9 m, and 16 m and fixed in 4% paraformaldehyde in PBS at 4°C overnight and decalcified with 0.5 M EDTA for 2 days. The cochleae were embedded in celloidin. Serial cross sections (8–10 µm) were prepared with a microtome (CM3050, Leica, Nussloch, Germany) and collected on microslides. The sections were stained with Hematoxylin and Eosin and then washed. The slides were mounted with regular mounting solution (glycerol) and examined under a microscope. Images at 10× and 25× magnification were taken from middle turns. The area of the spiral ligament and the SV was determined using ImageJ (version 1.47) from captured images.

## Results

### 1. Changes of EP magnitude during aging

**Figure 1:**
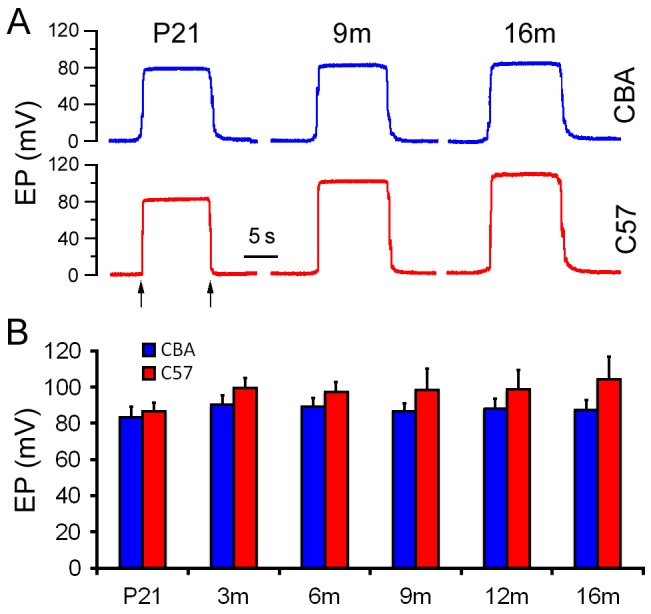
EP waveforms and magnitude change during aging. A. Representative waveforms of EP at three different ages. B. Mean and SD of EP magnitude at different time points during aging. Arrows indicate electrode penetrated into and withdrawn from the scala media.

The CBA and C57 mice are commonly used for the study of ARHL [27], [28]. Hearing threshold and EP are often measured to assess the functional status of the OC and the LW of the scala media. Both hearing threshold and EP of the two strains have been examined before [29], [30], [31]. We and others have shown that C57 mice display an onset and rapid progression of hearing loss starting from high frequencies as early as 3 m, and by 16 m profound hearing loss greater than 100 dB is observed across the frequency range tested [20]. In contrast, CBA mice exhibit minimal hearing loss (less than 20 dB) between P21 and 16 m. To determine the functional status of the LW during aging and to provide guidance for tissue collection for molecular biology studies, we measured basal turn EP at P21 and 3 m, 6 m, 9 m, 12 m, and 16 m. Seven animals (seven ears) from each age/strain were used. Fig. 1A shows some representative responses from such recordings. The EP response was a constant polarization with a magnitude of 80–100 mV when the electrode penetrated the endolymphatic spaces. The mean value of EP at different ages was determined and is presented in Fig. 1B. The EP magnitude of CBA mice did not display any significant change between P21 and 16 m (p>0.05 in all cases). In contrast, the EP magnitude of C57 mice exhibited a significant elevation at 3 m (p<0.05 between P21 and 3 m) and remained elevated between 3 m and 16 m. Thus, while the hearing thresholds of C57 mice began to deteriorate at high frequency from 3 m, the EP magnitude showed a paradoxical increase from 3 m. The elevation at 3 m and beyond is the result of progressive hair cell loss beginning at 3 m [20].

### 2. Morphological changes of the LW during aging

**Figure 2:**
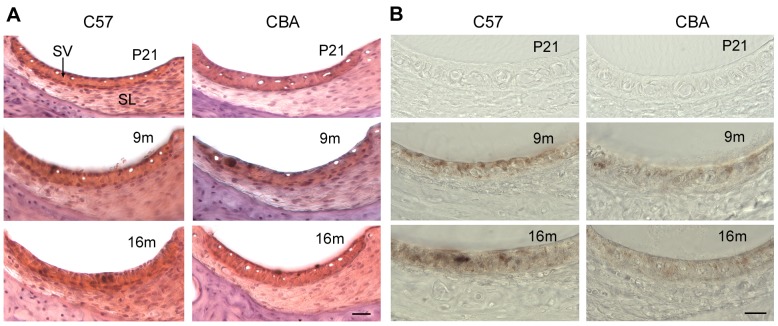
Cross sections of the LW at three different times points during age. Sections were obtained from cochlear middle turns at P21, 9 m, and 16 m. A. Cross sections stained with Hematoxylin and Eosin. Scale bars: 20 µm. B. Cross sections without any staining. Hyperpigmentation and reduction in the number of capillaries are apparent in the SV at 16 m.

We examined morphological changes of the LW during aging. Decrease in thickness of the SV and spiral ligament is often regarded as a sign of atrophy. As shown in Fig. 2, there are no apparent decrease in the thickness of the SV and the spiral ligament in C57 and CBA mice between P21 to 16 m. However, hyperpigmentation (Fig. 2B) and reduction in the number of capillaries (Fig. 2A) were observed in the SV at 16 m.

### 3. Differential expression of miRNAs in the LW during aging

**Figure 3:**
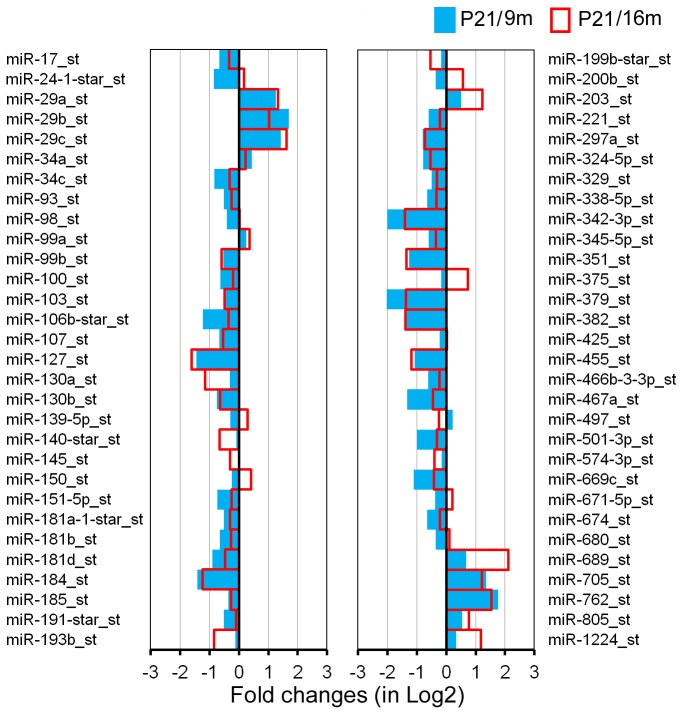
Differentially expressed miRNAs in the LW of CBA mice during aging. The X-axis relates Log2 transformed fold change in miRNA expression at 9 m (in blue) and 16 m (in red) compared to P21. Only those miRNAs whose levels were statistically different (p<0.05) from P21 were included.

**Figure 4:**
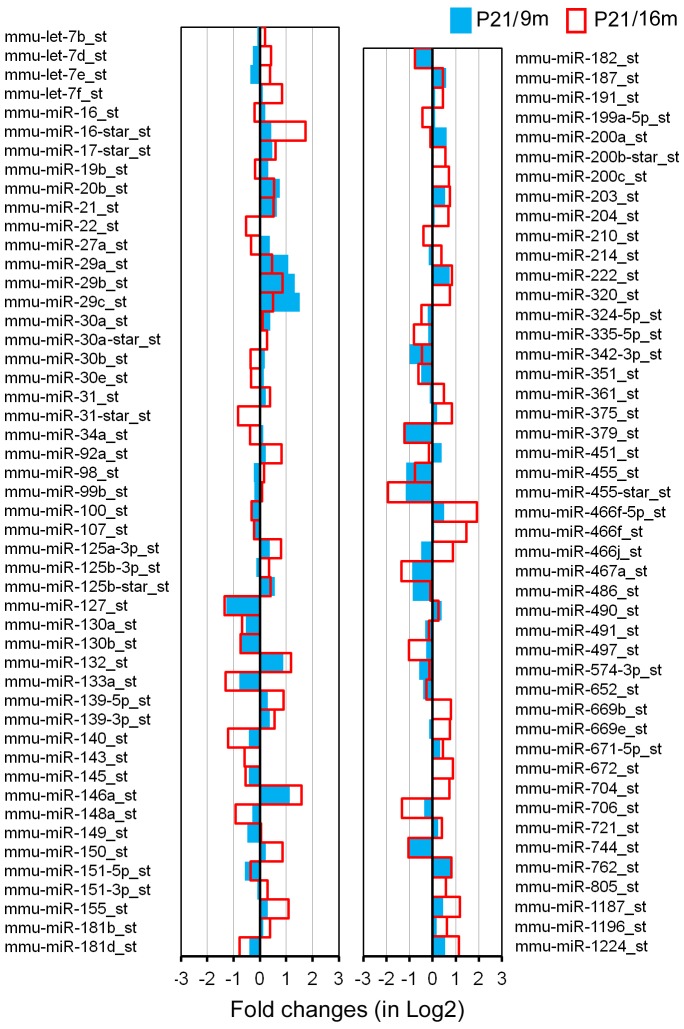
Differentially expressed miRNAs in the SV of C57 mice during aging. The X-axis relates Log2 transformed fold change in miRNA expression at 9 m (in blue) and 16 m (in red) compared to P21. Only those miRNAs which showed a statistically significant different in expression (p<0.05) compared to P21 are shown.

Total RNA isolated from LW of C57 and CBA mice was analyzed by miRNA microarray analysis that included 7,788 miRNA gene probe sets covering a variety of species. Our analyses were performed on all included mouse miRNAs (mmu-miRNAs). There were 609 probe sets for mmu-miRNAs. For comparison, the present study used the same criteria as those in our prior study of differentially expressed miRNAs in the OC [20]. The two criteria were: 1) the miRNA had to be “expressed” in each of the three repeats for each age group, and 2) the expression level of the miRNAs had to be significantly different from that of the youngest group (p<0.05, Student's t-test). miRNAs that met the two criteria are presented in Figs. 3 and 4.

We examined miRNA differential expression between P21 and 9 m as well as between P21 and 16 m in both strains. The rationale for selecting these time points is provided in our previous publication [20]. Selection of the same time points for collecting the OC and LW tissues made it possible to compare miRNA profiles between the two important structures in the inner ear. Fig. 3 depicts differential expression profiling of miRNAs from CBA mice between P21 and 9 m (in blue) as well as between P21 and 16 m (in red). A total of 60 miRNAs were identified as significantly differentially expressed during aging. Fifty miRNAs (83%) exhibited changes in the same direction (upregulation or downregulation) at the two different ages. Among the 50 miRNAs, downregulated miRNAs outnumbered upregulated miRNAs by a ratio of 3.5∶1 (39/11). Eleven miRNAs had their expression level downregulated by more than 50%, whereas 9 miRNAs were upregulated by more than twofold.

Fig. 4 presents differential expression profiles of miRNAs from C57 mice between P21 and 9 m (in blue) as well as between P21 and 16 m (in red). A total of 95 miRNAs were identified as significantly differentially expressed. Sixty-nine miRNAs (73%) showed changes in the same direction (upregulation or downregulation) at the two different ages. Among the 69 miRNAs, 33 were downregulated while 36 were upregulated, with a downregulation versus upregulation ratio of 0.9∶1. Thirty-four miRNAs had their expression level upregulated by more than twofold, whereas 7 miRNAs were downregulated by more than 50%. Comparison of miRNA expression profiles between C57 and CBA mice showed that the number of miRNAs indicated in aging of the LW of C57 mice is substantially greater than that of CBA mice during aging.

**Figure 5:**
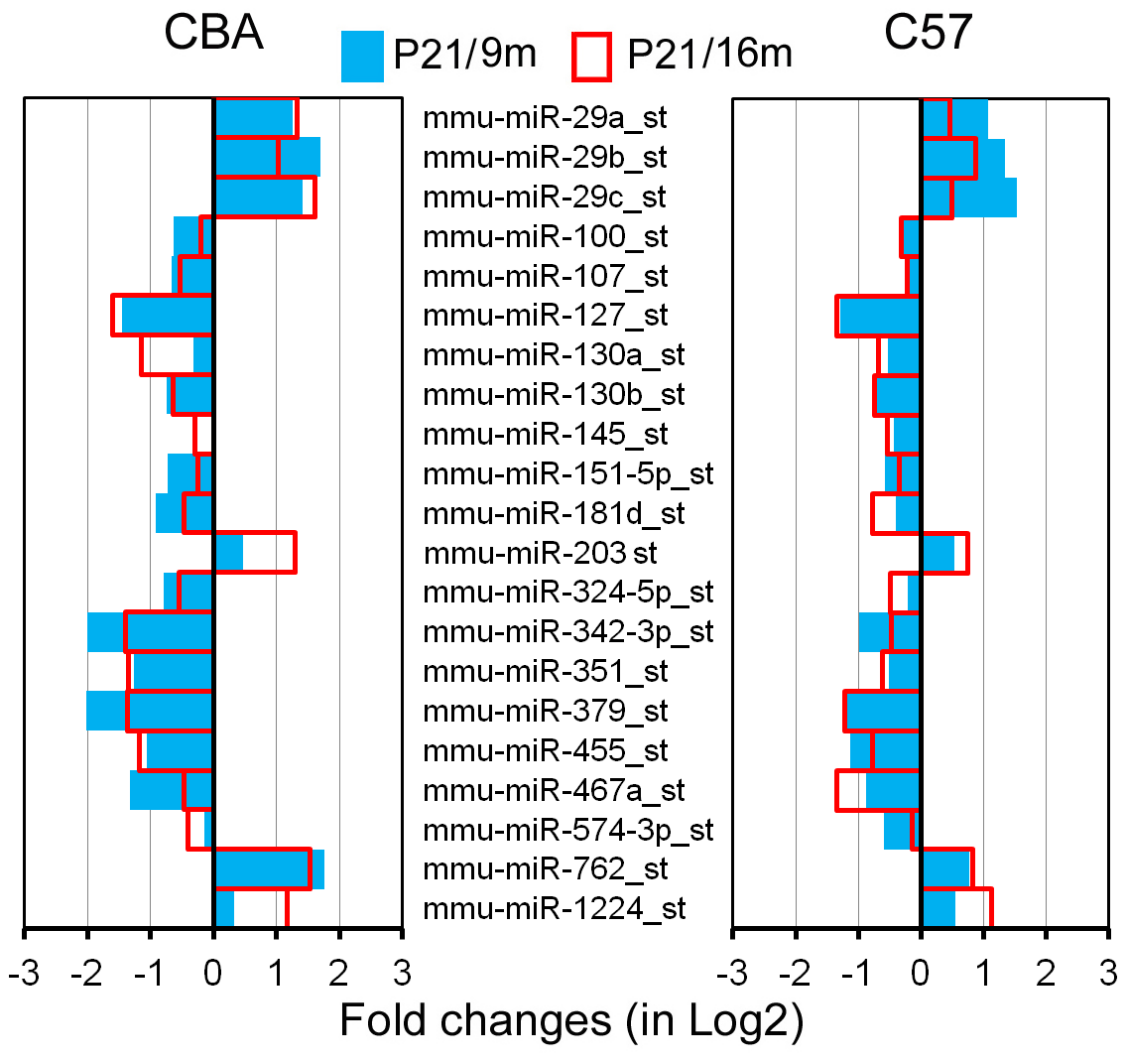
miRNAs that are commonly expressed in the LW of C57 and CBA mice during aging. Only those miRNAs that showed dynamic changes in the same direction (upregulated or downregulated) in both strains are included.

The early onset and rapid progression of hearing loss seen in C57 mice are due to *Ahl* gene mutation. As a result, the miRNA profiles for LW of C57 mice were expectedly different from those of CBA mice. However, C57 and CBA strains should share some common miRNAs associated with apoptosis and degeneration of LW. Fig. 5 depicts 21 common miRNAs that were differentially expressed during aging in both strains. Among the list, members of the miR-29 family, miR-203, miR-762, and miR-1224, showed upregulation, whereas members of the miR-107 family, miR-127 and miR-130a/b, miR-342-3p, miR-351, miR-379, miR-455, and miR-467a, were downregulated in both strains.

### 4. Temporal and spatial expression of four subsets of miRNAs in the LW

**Figure 6:**
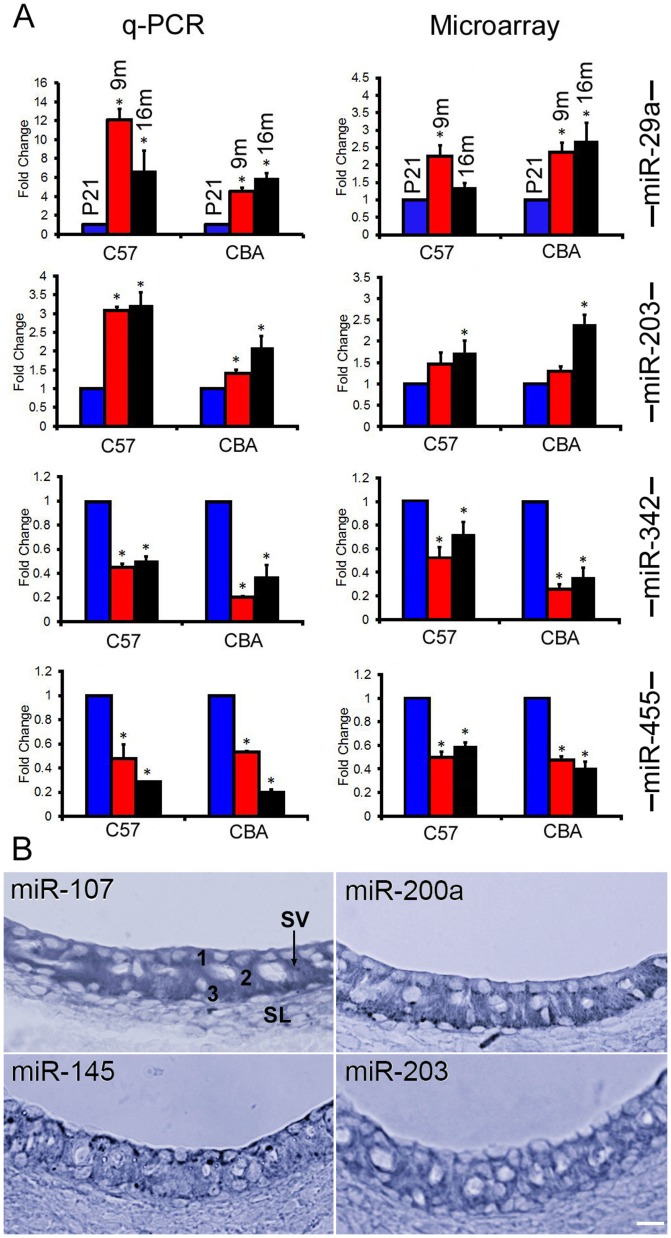
miRNA expression detected by q-PCR and *in situ* hybridization. A: Comparison of changes in miRNA expression detected by q-PCR versus microarray analyses for four miRNAs in the SV of C57 and CBA mice. Asterisks indicate statistically significant differences (p<0.05) compared to P21. Each q-PCR plot represents means from three repeats. B: Expression of four miRNAs in the LW of C57 mice using *in situ* hybridization technique. SV: Stria vascularis. SL: Spiral ligament. 1, 2, and 3 mark the three cell types in the SV (marginal, intermediate, and basal cells), respectively. Bar: 20 µm.

Q-PCR was used to validate some of the differentially expressed miRNAs indicated by the high-throughput microarray techniques. Two upregulated (miR-29a and miR-203) and two downregulated (miR-342 and miR-455) miRNAs shown in the microarray analyses were selected for q-PCR analysis. These miRNAs were selected since these four miRNAs are known to be involved in the pro-apoptotic and anti-apoptotic processes in other cells and diseases. miR-29a is also differentially expressed in the OC during aging in both strains of mice [20]. The fold change was determined from ΔΔC_T_ using the U6 as the internal control. Fig. 6A exhibits the dynamic changes of these miRNAs during aging by q-PCR assay. For comparison, changes in these four miRNAs from microarray analyses are also included. As shown, the expression levels of miR-342 and miR-455 in SV were significantly downregulated, while miR-29a and miR-203 were upregulated when compared to those of the P21 mice. The changes in miRNA expression determined by q-PCR were consistent with trends observed by microarray analyses.

We also examined the spatial expression of approximately 20 miRNAs in the LW of 3 m C75 mice using *in situ* hybridization. Four miRNAs showed strong expression in the SV (Fig. 6B). miR-107 and miR-200a were found in all three cell types in the SV. miR-203 was expressed in all cell types with weak expression in the marginal cells, while miR-145 was expressed in all cell types in the SV with strongest expression in the marginal cells.

### 5. Apoptosis-related gene expression in the LW

To elucidate the possible functional relevance of age-affected miRNAs, we next examined expression of genes that are associated with apoptosis and that were differentially expressed during aging. To determine which apoptosis-related genes are dynamically expressed in the LW during aging, a PCR-based apoptosis-related gene array (Table S1) was utilized. The array included 84 apoptosis-related genes in known extrinsic and intrinsic apoptotic pathways. Five stable housekeeping genes (Gusb, Hprt, Hsp90ab1, Gapdh, and Actb) were used as controls. We compared the expression levels of apoptosis-related genes in P21- and 9m-old C57 mice and in P21- and 16m-old CBA mice. Table 1 presents differentially expressed apoptosis-related genes in the LW with the fold change and p-value included in parenthesis. Atf5, Bid, Naip1, Birc2, Casp14, Dapk1, Mcl1, Nol3, Tnf, Cd40, Tnfsf10, and Zc3hcl were significantly upregulated, while Xiap, Birc5, Bok, and Casp3 were significantly downregulated in C57 mice. Most anti-apoptotic genes, such as Xiap and Birc5, were downregulated, while pro-apoptotic genes, such as Bid, Tnf and Tnfsf10, were upregulated in C57 mice. It is also apparent that more apoptosis-related genes were expressed in C57 mice than in CBA mice, despite the fact gene expression in C57 mice was assessed at 9 m (as opposed to 16 m for CBA mice).

**Table 1:** Differential expression of apoptosis-related genes in the LW of C57 and CBA mice.

Strain	Upregulated (Fold change, P value)	Downregulated (Fold change, P value)
C57BL/6 J: 9 m/P21	*Atf5 (3.053, 0.013)*	*Xiap (0.675, 0.034)*
	**Bid (3.239, 0.012)**	*Birc5 (0.305, 0.042)*
	*Naip1 (4.968, 0.015)*	**Bok (0.302, 0.033)**
	*BirC2 (2.281, 0.027)*	**Casp3 (0.593, 0.015)**
	**Casp14 (8.589, 0.004)**	
	*Dapk1 (3.697, 0.001)*	
	*Mcl1 (1.634, 0.0238)*	
	*Nol3 (5.795, 0.019)*	
	**Tnf (3.047, 0.025)**	
	**Cd40 (2.121, 0.008)**	
	**Tnfsf10 (1.886, 0.002)**	
	*Zc3hcl (2.440, 0.045)*	
CBA/J: 16 m/P21	*Dapk1 (4.385, 0.001)*	*Birc5 (0.103, 0.049)*
		**Casp3 (0.449, 0.034)**

Bold and Italics fonts represent pro-apoptotic and anti-apoptotic genes, respectively.

### 6. miRNAs and their target apoptosis-related mRNAs in the LW

In order to assess whether differential miRNA expression with age has an impact on putative target gene expression, we next examined the relationship between differentially expressed miRNAs and mRNAs. It is generally accepted that perfect miRNA seed region/target complementarity is predominantly associated with the destabilization of target mRNAs [32]. Accordingly, the level of a miRNA should be inversely correlated to the levels of its target gene transcripts. Differentially expressed miRNAs and their predicted target mRNAs were analyzed using TargetScan 6.2.

**Figure 7:**
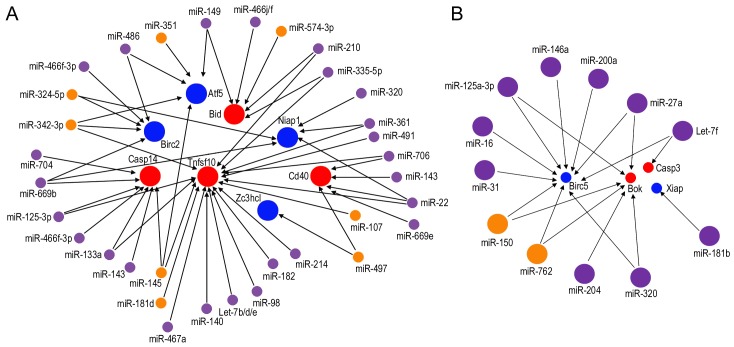
Network analysis of the relationship between differentially expressed miRNAs and mRNAs in the LW. A: Upregulated apoptosis-related genes predicted to be targeted by downregulated miRNAs. B: Downregulated apoptosis-related genes predicted to be targeted by upregulated miRNAs. Small circles represent downregulated miRNAs or mRNAs while large circles represent upregulated miRNAs or mRNAs. Purple and orange circles denote differentially expressed miRNAs in C57 and CBA mice, respectively. Red and blue circles represent pro-apoptotic and anti-apoptotic genes, respectively.

Our analyses identified 8 upregulated mRNAs (Atf5, Bid, Birc2, Casp14, Tnfsf10, Zc3hcl, Cd40, and Niap1) and 4 downregulated mRNAs (Birc5, Bok, Casp3, and Xiap) that contained binding sites for differentially expressed miRNAs identified in the LW of C57 mice. Fig. 7 presents the networks of potential interaction between the miRNAs and mRNAs. The coincident relationships that pro-apoptotic miRNAs regulate anti-apoptotic genes, and anti-apoptotic miRNAs regulate pro-apoptotic genes, are exhibited in the networks. For example, Tnfsf10, a critical pro-apoptotic gene whose expression was upregulated by 1.9-fold in the LW of C57 mice, is predicted to be regulated by two anti-apoptotic miRNAs, miR-145 and miR-107, which were downregulated at 9 m. Also, Birc5, an anti-apoptotic gene downregulated in both strains during aging, is predicted to be regulated by miR-200a, a known pro-apoptotic miRNA that was upregulated at 9 m in the LW of C57 mice.

## Discussion

This is the first study that examines the extent and specificity of miRNA expression in the LW of scala media during aging in two strains of mice. We detected 95 and 60 miRNAs that exhibited significant differential expression in the LW of C57 and CBA mice, respectively, with aging. Many of those differentially expressed miRNAs are predicted to target pathways and processes relevant to aging. Most miRNAs that were significantly upregulated are pro-apoptotic, while most of those downregulated are anti-apoptotic or pro-proliferative. Although the precise function of these individual miRNAs in regulating cellular senescence and aging in the LW is yet to be determined, it is conceivable that the underlying process and regulatory mechanism might involve a gradual and collective action of repression of miRNAs important for proliferation and differentiation, and concomitant enhancement of miRNAs that promote apoptosis during aging. Such changes of miRNA expression precede morphological and functional changes that can be detected.

In our previous study, we identified 111 and 71 miRNAs that exhibit differential expression in the OC of C57 and CBA mice, respectively [20]. Comparison of miRNA expression profiles in the OC and LW shows that several miRNAs are expressed in both tissues. These miRNAs include miR-29a/b, miR-100, miR-107, miR-130b, miR-193b, miR-343-3p, miR-351, and miR-455. miRNAs that were differentially expressed only in the LW during aging include miR-29c, miR-705, miR-99a, miR-127, miR-130a, miR-145, miR-151-5p, miR-379, miR-467a, and miR-574-3p. While miR-29c and miR-705 were upregulated, the rest were downregulated. Members of the miR-29 family (miR-29a/b/c) were significantly upregulated in the LW of both strains (Fig. 5). More than a two-fold increase of miR-29a/b was reported in the OC during onset and progression of ARHL [20]. This family is involved in cellular senescence and apoptosis in several cell lines, tissues, and organisms during aging [33]–[36]. miR-203, a transcriptional target of p53/TA-p73/p63 [37], is an anti-proliferative/pro-apoptotic miRNA involved in regulation of proliferation, differentiation, and apoptosis in several cell types (including epithelia and melanocyte) through p16/Rb and p53 pathways [38], [39]. It also targets E2F3 and ZBP-89, inducing cell cycle arrest by inhibiting p16 expression [40]. In melanocytes, miR-203 acts as a tumor suppressor by inducing senescence, and ABL1 is the target of miR-203, suggesting an anti-proliferative function of this miRNA [41]. miR-203 exhibited significant upregulation in the LW of both strains, and its expression was confirmed by both q-PCR and *in situ* hybridization (Figs. 6A, B). Since miR-203 is an anti-proliferative/pro-apoptotic miRNA and was upregulated, it is likely this miRNA is involved in degeneration of the SV.

It is interesting to observe the enhanced expression of miR-762 and miR-1224 in the LW. Although little is known about the role of these two miRNAs, two recent studies show that these two miRNAs are upregulated during infection and inflammatory responses. miR-762 has been found ubiquitously distributed in murine ocular tissues [42] and negatively regulates the expression of genes encoding the antimicrobial RNase7, the immunomodulator ST2, and the RhoGTP-binding protein Rab5a [43]. miR-1224 is highly expressed in mouse spleen, kidney, and lung [44]. Transfection of miR-1224 results in a decrease in basal tumour necrosis factor-α (TNF-α) promoter reporter gene activity and a downregulation of LPS-induced TNF-α mRNA in RAW264.7 cells [44]. If these two miRNAs are involved in inflammatory responses, it suggests that inflammation of the SV might be a component of degeneration in the SV during aging.

A number of miRNAs were downregulated in the LW during aging. These miRNAs include miR-107, miR-127, miR-130a/b, miR-145, miR-342, miR-351, miR-379, miR-455, and miR-467. These miRNAs have been detected in other tissues. Although a miRNA can have different roles and even opposite functions in different tissues, these miRNAs are, in general, pro-proliferative or anti-apoptotic. miR-127 is part of the miRNA signature that is upregulated in acute myeloid leukaemia [45] and in nodal diffuse large B-cell lymphomas [46]. miRNA-130a is oncogenic, contributing to colon tumorigenesis by regulating TGF-β/Smad signaling [47]. miR-351 is detected during myogenic progenitor cell differentiation and inhibits E2f3 expression, a key regulator of cell cycle progression and proliferation [48]. miR-379 is expressed in breast cancer, regulating interleukin-11 and Cyclin B1 [49]. The expression of miRNA-455 is detected in adrenal, ovarian granulosa, and model Leydig cell lines, and its expression is downregulated by trophic hormones or their second messenger, cyclic AMP [50]. miRNA-455 is also important for chondrogenesis [51]. miR-467a, an abundant member of the Sfmbt2 cluster, can promote cell proliferation. Finally, miR-467a can promote growth and survival of mouse ES cells [52].

Four miRNAs among the downregulated miRNAs in the LW deserve some discussion, since they may be related to function and maintenance of intermediate cells (melanocytes) in the SV. Intermediate cells play an important role for maintenance of endolymph and generation of EP. Mutations in melanocytes result in loss of EP and hair cells [53]–[55]. The four downregulated miRNAs are miR-107, miR-145, miR-342, and miR-455. miR-107, together with miR-15a/b, miR-16, miR-103, miR-195, miR-424, miR-497, miR-503, and miR-646, belongs to the miR-15-107 group. This cluster of miRNAs regulates gene expression involved in cell division, metabolism, and stress response [56], [57]. This group has been implicated in some age-related diseases, such as cardiovascular disease and neurodegenerative disease including Alzheimer's [58], [59]. miR-107 is expressed abundantly in brain tissue [60]. Our *in situ* hybridization showed miR-107 was expressed in the SV (Fig. 6B). Intermediate cells in the SV are derived from neural crest cells. Downregulation of miR-107 during aging in the SV is coincident with its anti-apoptotic function, suggesting that decline of its regulatory pathways might contribute to stria degeneration.

miR-145 was also detected in the SV by *in situ* hybridization (Fig. 6B). Previous studies demonstrated miR-145 is abundantly expressed in the smooth muscle tissues and critical for vascular smooth muscle cell differentiation [61], [62]. miR-145 expression is also found in the vascular wall of the capillaries in the SV [63], [64] and in the intermediate cells. Dynoodt et al. [65], [66] demonstrated that miR-145 is a key regulator in melanogenesis. The expression of miR-145 in melanocytes exhibits a negative relationship with the expression of Sox9, Mitf, Tyr, Trp1, Myo5a, Rab27a, and Fscn1. Downregulation of this miRNA during aging is consistent with its role in regulating vascular smooth muscle and melanocytes in the SV.

One of the putative targets of miR-455 is PAX6, which has been shown to play a critical role in the self-renewal and differentiation of neural stem cells. miR-455 is also detected in primary melanoma cell lines and metastatic melanomas [51], [67]–[69]. It is speculated that the loss of miR-455 and its subsequent effect on PAX6 expression may disrupt the normal progression of melanogenesis. Reduced expression of miR-455 was evident in our microarray analysis and q-PCR validation, and it might reflect a reduction of intermediate cells in the SV during aging. Similarly, miR-342 was also downregulated and has been shown to regulate melanogenesis [70].

Apoptosis-related q-PCR arrays are often used to analyze gene expression related to known apoptosis pathways. In the auditory system, such analyses have been used to examine gene expression after ototoxic drug treatment or after noise exposure [25], [71]. We combined apoptosis-related q-PCR array results with miRNA microarray analyses and bioinformatics approaches to explore potential miRNA-mRNA networks in the LW during aging. While the relationship between some of these miRNAs and mRNAs have been shown before in other tissues and cell lines, this is the first time that their relationship (Fig. 7) has been examined in the LW during aging.

Notably, our data revealed that expression of Tnfsf10 (upregulated by ∼2-fold) is likely influenced by several miRNAs including miR-107, miR-145, miR-342-3p, miR-491, miR-494, miR-182, and miR-467a. miR-107 and miR-145 were found to be expressed in the SV and downregulated during aging in both strains. Tnfsf10 is a pro-apoptotic cytokine that belongs to the tumor necrosis factor ligand family. It predominantly induces apoptosis in transformed and tumor cells [72], [73]. Tnfsf10 binds to several members of the TNF receptor superfamily and triggers the activation of MAPK8/JNK, caspase-8, and caspase-3. Tnfsf10 is upregulated in cochlear spiral ganglion cells in the early stage of salicylate-induced degeneration [25]. This suggests that TNF receptor families play an important role in cochlear degeneration by eliciting caspase-mediated cell death. Tnfsf10 is predicted to be directly targeted by miR-107 and miR-145, which were found to be expressed in the SV and downregulated during aging in both strains. miR-145 additionally targets the insulin receptor substrate-1 (IRS1), a factor in the insulin/insulin-like growth factor-1 (IGF-1) signaling pathway, the first established aging pathway [74].

Some other apoptosis-related genes identified in our study have been linked to degeneration and cell death in the inner ear. For example, BH3 interacting domain death agonist, or Bid, a member of the Bcl-2 family of cell death regulators and a mediator of mitochondrial pathway induced caspase-8, is a predicted target of miR-574-3p and miR-494. Bid is activated following enhanced caspase-8 activity in cisplatin-induced apoptosis of auditory cells *in vitro* through both the death receptor mechanisms and mitochondrial pathways of apoptosis [75]. Co-expression of Bid and its predicted miRNA regulators in both OC (our unpublished observation) and LW during aging indicates that regulation of Bid and its related signaling pathways are crucial during cochlear ARHL. Another example is Birc5, a member of the inhibitor of apoptosis gene family that encodes negative regulatory proteins that prevent apoptotic cell death. Birc5 was significantly downregulated during aging. Previous studies demonstrated an oto-protective role of Birc5 against ototoxic and noise insults [76], [77]. Birc5 is predicted to be regulated by miR-27b, miR-125b-3p, miR-150, miR-200a, miR-146a, miR-709, miR-705, and miR-762.

A number of issues deserve some discussion. First, the selection of the two mouse strains for the current study. We are fully aware that neither mouse strain shows a stria-originated ARHL. We used CBA mice as a model to identify miRNAs involved in physiological aging. C57 mice have an early onset and rapid progression of hearing loss due to *Ahl* gene mutation. This strain is not an ideal model to study molecular mechanism of aging in the LW. However, since C57 has a progressive hair cell loss during aging, it would be interesting to determine how degeneration of the OC would affect strial function and miRNA expression profile in this strain. Furthermore, we examined miRNA expression profiles of the OC during aging in CBA and C57 mice. Comparison of miRNA expression profiles between the OC and the LW during aging in the same strain and between the two strains would allow us to identify common pathways involved in the degeneration of the OC and LW during aging. Second, we observed an increase in EP magnitude in C57 mice during aging, which has never been reported before. Most studies that have examined EP magnitude change during aging often started at later stage (i.e., after 3 m) and therefore did not observe the change. Progressive hair cell loss is most likely responsible for the elevation in EP magnitude, although a delay in stria development could not be ruled out either. Hair cells have a standing (leak) current through mechanotransduction channels at rest [78] which reduces the voltage difference between scala media and scala tympani. Early hair cell loss diminishes the leak current and increases the resistance between the scala media and scala tympani, resulting the increase in EP. Third, we observed no significant reduction in EP magnitude during aging although some minor morphological changes were observed in the LW of C57 mice. However, significant change in miRNA expression was already detected. The fact that differential expression of miRNAs occurred well before morphological and functional changes in both strains of mice supports our previous conclusion that changes in miRNA expression precede morphological and functional changes [20]. Fourth, we showed that downregulated miRNAs outnumber upregulated miRNAs by a wide margin in the OC of both strains during aging in our previous study. This global decline of miRNA during aging was also observed in the LW of CBA mice and was consistent with the trend observed in other studies [79]. For example, in the aging brain, 85 miRNAs are downregulated, while only 8 are upregulated [79]. The vast majority of *C. elegans* miRNAs that are differentially expressed during aging are also downregulated [80]. Interestingly, the number of upregulated and downregulated miRNAs was essentially equal in the LW of C57 mice (Fig. 4). Therefore, it appears that there was a significant increase in upregulated miRNAs in C57 during aging. Two previous studies examining miRNAs in the aging brain and liver also reported predominant miRNA upregulation [81], [82]. It is important to note that different degrees of cochlear deterioration in two different strains may provide a possible explanation for the different results. Comparison of miRNA expression profiles between C57 and CBA mice showed that the number of miRNAs involved in the LW of C57 mice is significantly greater than in CBA mice during aging. This is consistent with the fact that the onset of hearing loss occurs much earlier and progresses more rapidly in C57 mice than in the CBA strain. Fifth, we are fully aware that miRNAs function as post-transcriptional regulators of gene expression that might only effect protein expression. We only examined target mRNA levels for proteins that are part of apoptosis-related pathways, and other effects have undoubtedly been missed. Although Western blotting can be used to examine protein expression, it was technically impractical to examine many proteins involved in apoptosis in these small tissue samples. Finally, aging is a multidimensional process that involves multiple molecular mechanisms and pathways. While apoptosis-related cell loss and atrophy maybe part of the mechanisms involved in aging, other mechanisms and pathways are also involved. The present study only examined genes associated with apoptosis. Future experiments will examine other pathways associated with aging.

The differential expression of many miRNAs during aging in the LW supports their involvement in a complex process of age-related degeneration of stria function that is not molecularly well understood. The pattern of altered miRNA expression we observe here suggests that multiple gene regulatory relationships are affected during aging. These findings point to novel functions for miRNAs in the molecular mechanisms of aging. The present work is the first step in an effort to elucidate the roles of miRNAs and their regulatory networks in age-related degeneration of the LW. It also lays the groundwork for future experiments that can explore whether suppression or overexpression of some miRNAs can slow the onset and progression of stria degeneration.

## Supporting Information

Table S1 **84 apoptosis related genes contained in the apoptosis RT^2^ Profiler PCR Array.**
(DOCX)
