# Supplementary material for: Identifying MicroRNAs Involved in Aging of the Lateral Wall of the Cochlear Duct
Source: PLoS One. 2014 Nov 18;9(11):e112857. doi: 10.1371/journal.pone.0112857 (PMC4236067; doi:10.1371/journal.pone.0112857)
Supplement: Table S1 — 84 apoptosis related genes contained in the apoptosis RT2 Profiler PCR Array. (DOCX) [file pone.0112857.s001.docx]

Table S1: 84 apoptosis related genes contained in the apoptosis RT² Profiler PCR Array

| Symbol |  |  | Description |
| --- | --- | --- | --- |
| Akt1 |  |  | Thymoma viral proto-oncogene 1 |
| Apaf1 |  |  | Apoptotic peptidase activating factor 1 |
| Api5 |  |  | Apoptosis inhibitor 5 |
| Atf5 |  |  | Activating transcription factor 5 |
| Bad |  |  | BCL2-associated agonist of cell death |
| Bag1 |  |  | Bcl2-associated athanogene 1 |
| Bag3 |  |  | Bcl2-associated athanogene 3 |
| Bak1 |  |  | BCL2-antagonist/killer 1 |
| Bax |  |  | Bcl2-associated X protein |
| Bcl10 |  |  | B-cell leukemia/lymphoma 10 |
| Bcl2 |  |  | B-cell leukemia/lymphoma 2 |
| Bcl2l1 |  |  | Bcl2-like 1 |
| Bcl2l10 |  |  | Bcl2-like 10 |
| Bcl2l2 |  |  | Bcl2-like 2 |
| Bid |  |  | BH3 interacting domain death agonist |
| Naip1 |  |  | NLR family, apoptosis inhibitory protein 1 |
| Naip2 |  |  | NLR family, apoptosis inhibitory protein 2 |
| Birc2 |  |  | Baculoviral IAP repeat-containing 2 |
| Birc3 |  |  | Baculoviral IAP repeat-containing 3 |
| Xiap |  |  | X-linked inhibitor of apoptosis |
| Birc5 |  |  | Baculoviral IAP repeat-containing 5 |
| Bnip2 |  |  | BCL2/adenovirus E1B interacting protein 2 |
| Bnip3 |  |  | BCL2/adenovirus E1B interacting protein 3 |
| Bnip3l |  |  | BCL2/adenovirus E1B interacting protein 3-like |
| Bok |  |  | BCL2-related ovarian killer protein |
| Card10 |  |  | Caspase recruitment domain family, member 10 |
| Nod1 |  |  | Nucleotide-binding oligomerization domain containing 1 |
| Card6 |  |  | Caspase recruitment domain family, member 6 |
| Casp1 |  |  | Caspase 1 |
| Casp12 |  |  | Caspase 12 |
| Casp14 |  |  | Caspase 14 |
| Casp2 |  |  | Caspase 2 |
| Casp3 |  |  | Caspase 3 |
| Casp4 |  |  | Caspase 4, apoptosis-related cysteine peptidase |
| Casp6 |  |  | Caspase 6 |
| Casp7 |  |  | Caspase 7 |
| Casp8 |  |  | Caspase 8 |
| Casp9 |  |  | Caspase 9 |
| Cflar |  |  | CASP8 and FADD-like apoptosis regulator |
| Cidea |  |  | Cell death-inducing DNA fragmentation factor, alpha subunit-like effector A |
| Cideb |  |  | Cell death-inducing DNA fragmentation factor, alpha subunit-like effector B |
| Cradd |  |  | CASP2 and RIPK1 domain containing adaptor with death domain |
| Dad1 |  |  | Defender against cell death 1 |
| Dapk1 |  |  | Death associated protein kinase 1 |
| Dffa |  |  | DNA fragmentation factor, alpha subunit |
| Dffb |  |  | DNA fragmentation factor, beta subunit |
| Tsc22d3 |  |  | TSC22 domain family, member 3 |
| Fadd |  |  | Fas (TNFRSF6)-associated via death domain |
| Fas |  |  | Fas (TNF receptor superfamily member 6) |
| Fasl |  |  | Fas ligand (TNF superfamily, member 6) |
| Hells |  |  | Helicase, lymphoid specific |
| Il10 |  |  | Interleukin 10 |
| Lhx4 |  |  | LIM homeobox protein 4 |
| Ltbr |  |  | Lymphotoxin B receptor |
| Mcl1 |  |  | Myeloid cell leukemia sequence 1 |
| Nfkb1 |  |  | Nuclear factor of kappa light polypeptide gene enhancer in B-cells 1, p105 |
| Nme5 |  |  | Non-metastatic cells 5, protein expressed in (nucleoside-diphosphate kinase) |
| Nol3 |  |  | Nucleolar protein 3 (apoptosis repressor with CARD domain) |
| Pak7 |  |  | P21 protein (Cdc42/Rac)-activated kinase 7 |
| Pim2 |  |  | Proviral integration site 2 |
| Polb |  |  | Polymerase (DNA directed), beta |
| Prdx2 |  |  | Peroxiredoxin 2 |
| Pycard |  |  | PYD and CARD domain containing |
| Ripk1 |  |  | Receptor (TNFRSF)-interacting serine-threonine kinase 1 |
| Rnf7 |  |  | Ring finger protein 7 |
| Sphk2 |  |  | Sphingosine kinase 2 |
| Tnf |  |  | Tumor necrosis factor |
| Tnfrsf10b |  |  | Tumor necrosis factor receptor superfamily, member 10b |
| Tnfrsf11b |  |  | Tumor necrosis factor receptor superfamily, member 11b (osteoprotegerin) |
| Tnfrsf1a |  |  | Tumor necrosis factor receptor superfamily, member 1a |
| Cd40 |  |  | CD40 antigen |
| Tnfsf10 |  |  | Tumor necrosis factor (ligand) superfamily, member 10 |
| Tnfsf12 |  |  | Tumor necrosis factor (ligand) superfamily, member 12 |
| Cd40lg |  |  | CD40 ligand |
| Cd70 |  |  | CD70 antigen |
| Traf1 |  |  | Tnf receptor-associated factor 1 |
| Traf2 |  |  | Tnf receptor-associated factor 2 |
| Traf3 |  |  | Tnf receptor-associated factor 3 |
| Trp53 |  |  | Transformation related protein 53 |
| Trp53bp2 |  |  | Transformation related protein 53 binding protein 2 |
| Trp53inp1 |  |  | Transformation related protein 53 inducible nuclear protein 1 |
| Trp63 |  |  | Transformation related protein 63 |
| Trp73 |  |  | Transformation related protein 73 |
| Zc3hc1 |  |  | Zinc finger, C3HC type 1 |
